# Surgical necrotizing enterocolitis but not spontaneous intestinal perforation is associated with adverse neurological outcome at school age

**DOI:** 10.1038/s41598-020-58761-6

**Published:** 2020-02-11

**Authors:** Alexander Humberg, Juliane Spiegler, Mats Ingmar Fortmann, Michael Zemlin, Janina Marissen, Isabelle Swoboda, Tanja K. Rausch, Egbert Herting, Wolfgang Göpel, Christoph Härtel, The German Neonatal Network (GNN), Christian Wieg, Angela Kribs, Axel von der Wense, Ursula Weller, Thomas Höhn, Dirk M. Olbertz, Ursula Felderhoff-Müser, Rainer Rossi, Norbert Teig, Friedhelm Heitmann, Susanne Schmidtke, Bettina Bohnhorst, Matthias Vochem, Holger Michel, Jens Möller, Joachim G. Eichhorn, Jürgen Wintgens, Ralf Böttger, Mechthild Hubert, Michael Dördelmann, Georg Hillebrand, Claudia Roll, Reinhard Jensen, Mario Rüdiger, Julia Sandkötter, Stefan Schäfer, Thomas Schaible, Axel Franz, Malik Aydin, Silke Ehlers, Claudius Werner, Thorsten Orlikowsky, Hubert Gerleve, Katja Schneider, Claudius Werner, Kai Böckenholt, Knud Linnemann, Dirk Müller, Corinna Gebauer, Florian Guthmann, Jochen Reese, Roland Haase, Stephan Seeliger, Helmut Küster, Roland Hentschel, Thorsten Körner, Thomas Brune, Andreas Müller, Thomas Frank, Martin Andree Berghäuser, Kristin Dawczynski

**Affiliations:** 1 Department of Paediatrics, University Hospital of Schleswig-Holstein, Campus Lübeck, Lübeck, Germany; 2 Department of Paediatrics and Neonatology, Saarland University Medical Center, Homburg/Saar, Germany; 3 Institute of Medical Biometry and Statistics, University of Lübeck, University Hospital of Schleswig-Holstein, Campus Lübeck, Lübeck, Germany; 4 Children’s Hospital Aschaffenburg-Alzenau, Aschaffenburg, Germany; 5 University Hospital of Cologne, Cologne, Germany; 6 Children’s Hospital Hamburg-Altona, Hamburg, Germany; 7 Evangelical Klinikum Bethel, Bielefeld, Germany; 8 University of Düsseldorf, Düsseldorf, Germany; 9 Klinikum Südstadt Rostock, Rostock, Germany; 10 University Hospital of Essen, Essen, Germany; 11 Vivantes Klinikum Neukölln, Berlin, Germany; 12 University Hospital, Bochum, Germany; 13 Klinikum Dortmund, Dortmund, Germany; 14 Asklepios Hospital Hamburg-Barmbek, Hamburg-Barmbek, Germany; 15 Hannover Medical School, Hannover, Germany; 16 Olgahospital Stuttgart, Stuttgart, Germany; 17 Krankenhaus Barmherzige Brüder, Regensburg, Germany; 18 Saarbrücken General Hospital, Saarbrücken, Germany; 19 Klinikum Leverkusen gGmbH, Leverkusen, Germany; 20 Hospital Mönchengladbach, Mönchengladbach, Germany; 21 Universitatsklinikum Magdeburg, Magdeburg, Germany; 22 DRK Children’s Hospital, Siegen, Germany; 23 Diakonissen Hospital Flensburg, Flensburg, Germany; 24 Hospital Itzehoe, Itzehoe, Germany; 25 University Witten-Herdecke, Datteln, Germany; 26 Westküstenklinikum Heide, Heide, Germany; 27 University Hospital Carl Gustav Carus, Dresden, Germany; 28 University Hospital of Münster, Münster, Germany; 29 Children’s Hospital (Städtisches Klinikum) Nürnberg, Nürnberg, Germany; 30 University Medical Center Mannheim, Mannheim, Germany; 31 University of Tübingen, Tübingen, Germany; 32 HELIOS Children’s Hospital Wuppertal, Witten/Herdecke University, Wuppertal, Germany; 33 Bürgerhospital Frankfurt, Frankfurt, Germany; 34 Helios Klinik Schwerin, Schwerin, Germany; 35 University of Aachen, Aachen, Germany; 36 Christophorus Kliniken Coesfeld, Coesfeld, Germany; 37 GFO Hospitals Bonn, Bonn, Germany; 38 Children’s Hospital, University of Halle, Halle/Saale, Germany; 39 Children’s Hospital of the City of Cologne, Köln, Germany; 40 University of Greifswald, Greifswald, Germany; 41 Klinikum Kassel, Kassel, Germany; 42 University of Leipzig, Leipzig, Germany; 43 Kinderkrankenhaus auf der Bult, Hannover, Germany; 44 Children’s Hospital, Eutin, Germany; 45 University of Halle, Halle, Germany; 46 Klinikum St. Elisabeth, Neuburg/Donau, Germany; 47 Georg-August-University Göttingen, Göttingen, Germany; 48 University Hospital Freiburg, Freiburg, Germany; 49 Klinikum Links der Weser GmbH, Bremen, Germany; 50 Klinikum Lippe GmbH, Detmold, Germany; 51 University Hospital Freiburg, Freiburg, Germany; 52 St. Franziskus-Hospital Münster, Münster, Germany; 53 Florence-Nightingale Krankenhaus, Düsseldorf, Germany; 54 University Hospital Jena, Jena, Germany

**Keywords:** Risk factors, Infant necrotizing enterocolitis, Neonatal brain damage, Quality of life

## Abstract

Gastrointestinal complications during the neonatal period, i.e. necrotizing enterocolitis (NEC) and spontaneous intestinal perforation (SIP), are associated with adverse short-term outcome in very-low-birthweight infants (VLBWI, <1500 g birth weight). However, little is known about the neurological outcome of survivors at school age. We analysed data of 2241 infants followed-up at the age of 6 years. To determine the effect of NEC and SIP on cognitive outcome in consideration of other important confounding factors, we used multivariable logistic regression models. In addition, infants with surgical diagnosis of NEC (n = 43) or SIP (n = 41) were compared to NEC (n = 43) or SIP (n = 41) negative controls using Mahalanobis distance matching. Infants with a history for NEC had a three times increased risk (RR 3.0 [1.8–4.2], p < 0.001) to develop IQ scores <85 while history of surgical SIP did not increase the relative risk for lower IQs at school age (RR 1.0 [0.4–2.1], p = 1.000). In a matched-cohort analysis, we confirmed that infants with surgical NEC had lower mean IQ results than unaffected controls (±SD) (85±17 vs. 94±14, p = 0.023) while no differences were found for history of SIP. Our results reflect that the different aetiology and inflammatory extent of NEC and SIP may lead to disparate neurodevelopment trajectories. Hence, our data suggest a potential role of early gut-brain axis distortion in infants with NEC which needs to be further explored.

## Introduction

Necrotizing enterocolitis (NEC) and spontaneous intestinal perforation (SIP) are typical gastrointestinal complications in very-low-birthweight infants (VLBWI) and have a remarkable impact on mortality and long-term morbidity in this vulnerable group^1–4^. In VLBWI, NEC mainly occurs during day 14–28 of life^5^ and seems to have multifactorial facets including genetic predisposition, intestinal immaturity, inflammation, oxidative stress, ischemia, nutritional aspects and gut dysbiosis^2,6,7^. SIP usually occurs in the first 14 days of life^8^ and is mainly associated with extreme prematurity, use of non-steroidal and steroidal anti-inflammatory drugs and prolonged evacuation of meconium^9,10^. Recently, we noted an increased SIP risk in infants <25 weeks of gestation who were primarily managed with less invasive respiratory care^3^.

Epidemiological data of infants with NEC or SIP show delayed neurodevelopment in early childhood^2,9,11^, but little is known about outcome at school age. Roze *et al*. found an increased rate of reduced intelligence quotient (IQ) and motor testing scores among children at school age who suffered from NEC or SIP^11^ compared to controls without these complications. However, the authors did not adjust for confounding variables such as gestational age or maternal education level and did not differentiate between NEC and SIP.

Despite a potential clinical overlap between NEC and SIP, e.g. succus entericus-induced peritonitis, our study is based on the assumption that NEC and SIP are distinct entities with different pathophysiology and histopathological findings^12–15^. We here hypothesize that NEC - as a systemic “inflammatory disease” - and SIP - as a local gut disease – have different impact on long-term neurodevelopmental trajectories. The objective of our study was to evaluate the effect of NEC and SIP requiring surgery on intelligence and development of cerebral palsy at 6 years of age in a large cohort of very-low-birthweight infants from the German Neonatal Network.

## Results

### Cohort characteristics

**Figure 1:**
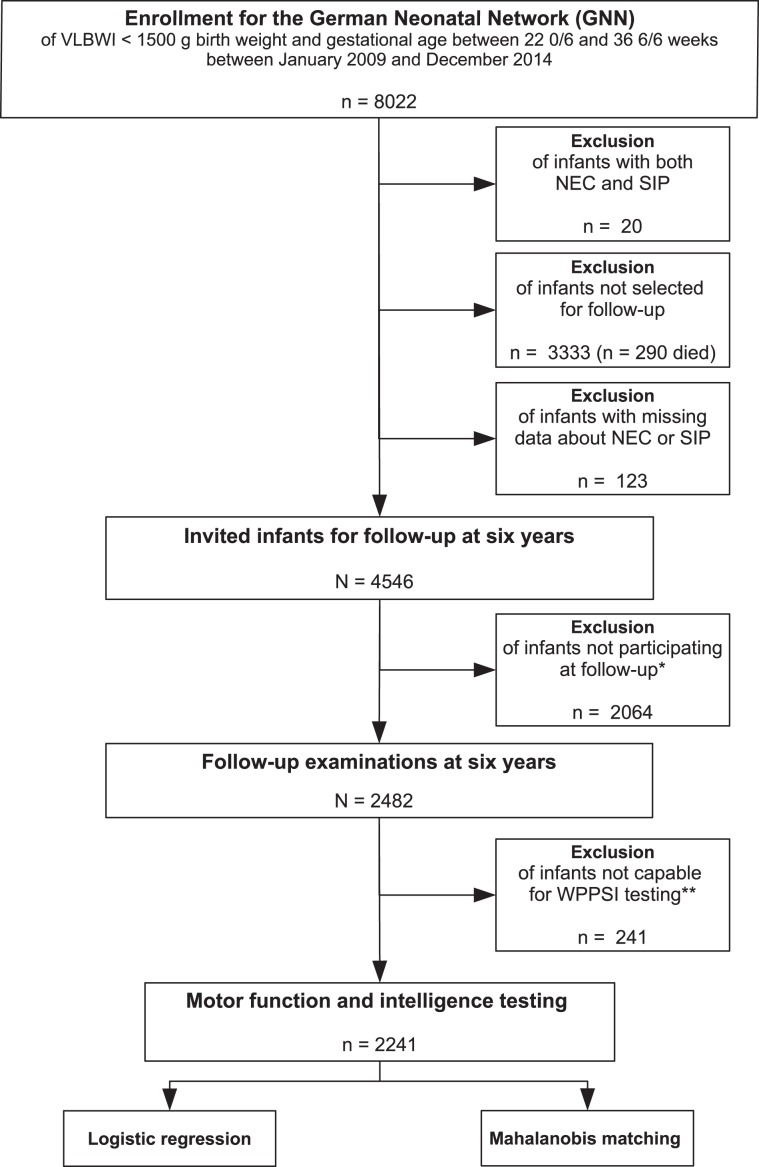
Enrollment, in- and exclusion for analysis of neurologic and motor development at the age of 6 years. In- and exclusion for motor function and intelligence testing in 6 year old children born as VLBWI; *Reasons for not participating the follow-up assessment despite selection were: no current contact data available n = 1349, parents declined invitation for follow-up n = 519, parents were interested to participate but were not available at suggested follow-up dates n = 131, no-show despite arranged follow-up assessment n = 65; **Reasons for no WPPSI assessment included: WPPSI or other cognitive test within 12 months n = 161, language problems n = 24, child not motivated n = 39, serious disorder not related to prematurity (e.g. trisomy 21) n = 7, other reasons n = 10.

In total, 8022 VLBWI were born and discharged in the German Neonatal Network between 2009 and 2014 (Fig. 1). Infants who suffered from both NEC and SIP were excluded (n = 20). Due to aspects concerning study coordination and organization, 3333 infants were not invited for follow-up. In 123 cases, the diagnosis of NEC or SIP was unknown. The remaining 4546 infants were invited for follow-up. In this group, 177 (2.2%) had a history of NEC while 123 (1.5%) infants had previously suffered from SIP. Of the invited 4546 infants, 2482 infants were tested for intelligence and motor function at the age of 6 years (Fig. 1). Baseline characteristics of the cohort at 6 years are described in Table 1. 241/2482 infants were not capable to perform a *Wechsler preschool and primary scale of intelligence* test (WPSSI-III, German edition) due to (a) WPPSI or other cognitive test performed within the last 12 months (n = 161), (b) language problems (n = 24), (c) lack of motivation (n = 39), (d) serious disorder not related to prematurity (e.g. trisomy 21) (n = 7) and (e) other reasons (n = 10). These infants had a significant lower gestational age (28.4 vs. 28.9 weeks GA, p < 0.001), birth weight (917 vs. 1163 g, p < 0.001) and an increased rate of neonatal complications including SIP (4.1 vs. 1.7%, p = 0.003) as compared to infants who performed WPPSI (Supplementary Table 1). Final analysis was based on a cohort of 2241 infants including 43 VLBWI (1.9%) with a history of surgical NEC and 41 (1.8%) who had previously suffered from surgical SIP. 306 VLBWI (13.7%) had an IQ < 85 on follow-up at school age.

**Table 1:** Clinical characteristics of VLBWI with an intraoperative diagnosis of NEC or SIP during admission and at follow-up at 6 years.

Characteristics	VLBWI cohort 2009–2014 n = 8002						Cohort who were followed-up at age 6 years n = 2241					
	NEC n = 177		SIP n = 123		No NEC and no SIP n = 7702		NEC n = 43		SIP n = 41		No NEC and no SIP n = 2157	
Gestational age (SD) [weeks]	26.3 (2.4)		25.6 (2.1)		29.1 (3.1)		26.1 (2.2)		25.4 (1.9)		29.1 (3.7)	
Birth weight (SD) [g]	810 (268)		749 (251)		1124 (459)		764 (256)		749 (260)		1205 (705)	
Head circumference (birth) [cm]	23.5 (2.6)		22.9 (2.5)		25.9 (2.7)		23.2 (2.5)		23.0 (2.5)		25.5 (2.8)	
Head circumference (discharge) [cm]	32.7 (4.1)		33.3 (3.5)		32.6 (2.8)		33.0 (2.5)		34.1 (2.2)		32.9 (2.4)	
Length (birth) [cm]	33.1 (3.8)		32.5 (3.6)		36.6 (4.1)		32.5 (3.9)		32.5 (3.8)		36.1 (4.3)	
Length (discharge) [cm]	46.7 (6.9)		46.8 (6.0)		45.6 (4.1)		47.1 (4.7)		48.1 (4.3)		46.0 (3.5)	
	**n (%)**	**95% CI**	**n (%)**	**95% CI**	**n (%)**	**95% CI**	**n (%)**	**95% CI**	**n (%)**	**95% CI**	**n (%)**	**95% CI**
Female Gender	83 (46.9)	39.6–54.2	41 (33.3)	25.5–42.0	3809 (49.5)	48.3–50.6	25 (58.1)	43.3–72.0	13 (31.7)	19.1–46.8	1082 (50.2)	48.0–52.3
Multiple birth	62 (35.0)	28.3–42.3	43 (35.0)	27.0–43.7	2567 (33.3)	32.3–34.4	15 (34.9)	22.0–49.7	19 (46.3)	31.8–61.4	763 (35.4)	33.4–37.4
Antenatal steroids	147 (83.1)	77.0–88.0	106 (86.2)	79.3–91.3	6721 (87.3)	86.5–88.0	35 (81.4)	67.9–90.8	38 (92.7)	81.7–97.9	1819 (84.3)	82.7–85.8
Small for gestational age	34 (19.2)	13.9–25.5	24 (19.5)	13.3–27.2	1410 (18.3)	17.4–19.2	8 (18.6)	9.2–32.1	9 (22.0)	11.5–36.2	368 (17.0)	15.5–18.7
ICH	72 (40.7)	33.6–48.0	54 (43.9)	35.4–52.7	1152 (15.0)	14.2–15.8	16 (37.2)	24.0–52.1	17 (41.5)	27.4–56.7	280 (13.0)	11.6–14.5
PVL	21 (11.9)	7.7–17.2	7 (5.7)	2.6–10.8	205 (2.7)	2.3–3.0	3 (7.0)	2.0–17.5	0	0	38 (1.8)	1.2–2.4
Neurosurgery	7 (4.0)	1.8–7.6	7 (5.7)	2.6–10.8	119 (1.5)	1.3–1.8	0	0	2 (4.9)	1.0–14.7	20 (0.9)	0.6–1.4
BPD	95 (53.7)	46.3–60.9	50 (40.7)	32.3–49.5	1060 (13.8)	13.0–14.6	18 (41.9)	28.0–56.7	14 (34.1)	21.1–49.3	309 (14.3)	12.9–15.9
European origin	142 (80.2)	74.0–85.5	94 (76.4)	68.4–83.2	6332 (82.2)	81.3–83.1	35 (81.4)	67.9–90.1	31 (75.6)	61.0–86.7	1803 (83.6)	82.0–85.1
Maternal education > 10 years^Ω^	n.a.	n.a.	n.a.	n.a.	n.a.	n.a.	16 (37.2)	24.0–52.1	18 (43.9)	27.6–57.3	900 (41.7)	39.6–43.8

Baseline characteristics of VLBWI of primary GNN cohort born and discharged between 2009 and 2014 and who were followed-up at age 6 years stratified by NEC or SIP. Data are given as n(%) and 95% CI if not mentioned; percentages are given as column percentages. Ω data about maternal education were collected at follow-up.

### NEC and SIP

Compared to infants without history of NEC, infants with surgical NEC in our cohort were born at lower gestational ages (26.3 vs. 29.1 weeks GA), lower birth weight (810 g vs. 1124 g) and received less antenatal steroids (83.1 vs. 89.8%) (see Table 1). VLBWI with NEC showed increased frequencies for intracerebral haemorrhage (ICH) (40.7 vs. 15.4%), periventricular leukomalacia (PVL) (11.9 vs. 2.7%), neurosurgery (4.0 vs. 1.6%) and bronchopulmonary dysplasia (BPD) (53.7 vs. 14.1%). VLBWI with surgical SIP were also born at lower gestational ages (25.6 vs. 29.1 weeks GA) and with lower birth weight (749 g vs. 1124 g) compared to non-SIP infants. SIP patients were more often male than female (66.7 vs. 33.3%) and of non-European ethnicity (23.0 vs. 14.7%). ICH (43.9 vs. 15.4%), PVL (5.7 vs. 2.7%), BPD (41.0 vs. 14.1%) were also increased in the SIP group.

### Neurodevelopmental outcome

To account for several confounding risk factors leading to adverse neurodevelopmental outcome as for example gestational age or other short-term complications, we performed multivariable logistic regression models and multidimensional Mahalanobis distance matching.

The multivariable logistic regression analysis revealed that infants with a history for NEC were associated with IQ scores <85 (OR 4.3 [2.1–8.8], p < 0.001) while history of surgical SIP was not associated with lower IQs at school age (OR 1.0 [0.4–2.6], p = 1.000) (see Supplementary Table 3). We calculated the relative risks from logistic regression by using the method described by Zhang *et al*.^16^. Here, infants with NEC showed up with a three times increased risk for IQ results < 85 (RR 3.0 [1.8–4.2], p < 0.001) (see Table 2). We matched history of NEC (n = 43) vs. controls (n = 43) and history of SIP (n = 41) vs. controls (n = 41) for different clinical parameters (gestational age, ICH, PVL, European ethnicity, BPD, female gender, antenatal administration of steroids, and highest maternal education level) using Mahalanobis distance. Baseline data about matched groups are given in Supplementary Table 2. SIP infants were born at significantly lower GA (25.4 ± 1.9 vs. 27.7 ± 2.2 weeks GA, p < 0.001) with lower birth weight (749 ± 260 vs 917 ± 309 g, p < 0.001), length (32.5 ± 3.8 vs. 34.7 ± 4.4, p = 0.021) and head circumference (23.0 ± 2.5 vs. 24.8 ± 2.6 cm, p = 0.003) than controls.

**Table 2:** Relative risks of factors influencing neurodevelopmental outcome.

Independent variable	Intelligence quotient <85			Cerebral palsy		
	RR (95% CI)	p-value	p-value§	RR (95% CI)	p-value	p-value§
Birth weight	1.0 (1.0–1.0)	0.871	1.000	1.0 (1.0–1.0)	0.421	1.000
Gestational age (per week)	0.9 (0.8–1.0)	0.013	0.224	0.8 (0.7–1.1)	0.066	1.000
Female gender	0.7 (0.8–0.9)	0.016	0.256	0.7 (0.4–1.1)	0.120	1.000
ICH ≥ grade 3 and PVL	2.7 (2.0–3.6)	<0.001	<0.001	5.2 (4.4–6.0)	<0.001	<0.001
Neurosurgery	2.8 (1.4–4.5)	<0.001	0.005	2.4 (1.2–3.8)	<0.001	0.003
European origin	1.7 (1.5–1.9)	<0.001	<0.001	1.3 (0.9–1.7)	0.226	1.000
Administration of surfactant	1.0 (0.7–1.3)	0.861	1.000	1.2 (0.6–2.0)	0.602	1.000
BPD	1.9 (1.3–2.7)	<0.001	0.015	1.6 (1.0–2.4)	0.020	0.411
SGA	1.9 (1.3–2.7)	0.001	0.019	1.3 (0.6–2.6)	0.431	1.000
Maternal education level	0.5 (0.3–0.6)	<0.001	<0.001	1.1 (0.7–1.5)	0.808	1.000
NEC	3.0 (1.8–4.2)	<0.001	<0.001	1.3 (0.5–3.1)	0.367	1.000
SIP	1.0 (0.4–2.1)	0.985	1.000	1.2 (0.4–2.7)	0.909	1.000

Relative risk (RR) and corresponding 95% confidence intervals of postnatal factors influencing neurodevelopmental outcome deriving from logistic regression (controlled for birth weight, gestational age (per weeks), female gender, ICH ≥ grade 3 and PVL, neurosurgery, European origin, administration of surfactant, BPD, SGA, and maternal education level (coded as maternal school attendance > 10 years)); ^§^Bonferroni-Holm correction (corrected for each model), significant findings (p < 0.05) are given in bold.

**Figure 2:**
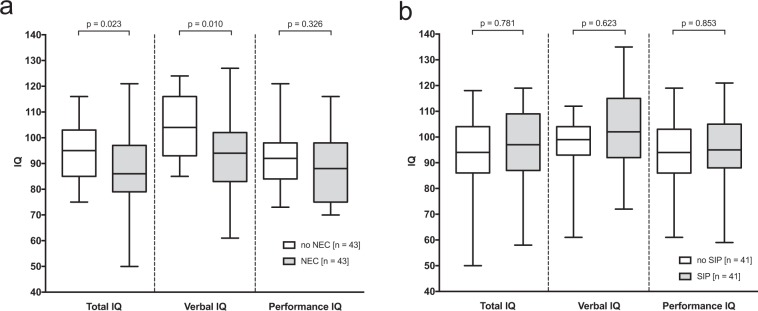
**(a**) Total, verbal and performance intelligence quotient scores in infants with and without NEC. Boxplot of IQ scores (total, verbal, performance) for matched infants with history of NEC and without a history of NEC. p-values are derived from T-Test. (**b**) Total, verbal and performance intelligence quotient scores in infants with and without SIP. Boxplot of IQ scores (total, verbal, performance) for matched infants with history of SIP and without a history of SIP. p-values are derived from T-Test.

In this matched model we confirmed that infants with surgical NEC had lower mean IQ results than unaffected controls (±SD) (85 ± 17 vs. 94 ± 14, p = 0.023) (Table 3, Fig. 2a). There were no differences between SIP patients and controls regarding cognitive impairments (93 ± 17 vs. 91 ± 21, p = 0.831) (Fig. 2b).

**Table 3:** Outcome characteristics of matched VLBWI at 6-year follow-up.

Characteristics	NEC			SIP		
	No n = 43	Yes n = 43	p-value	No n = 41	Yes n = 41	p-value
Weight [kg]	18.4 (3.0)	17.4 (3.4)	0.104	18.5 (3.6)	17.7 (3.2)	0.347
Head circumference [cm]	50.1 (2.1)	48.8 (2.0)	**0.004**	50.4 (2.3)	49.2 (2.4)	**0.011**
Length [cm]	113.0 (5.5)	110.0 (6.4)	**0.024**	111.6 (6.3)	110.9 (6.7)	0.633
IQ (mean, SD)	94 (14)	85 (17)	**0.023**	93 (17)	91 (21)	0.831
Cerebral palsy	3 (7.3)	6 (14.6)	0.289	4 (10.3)	5 (13.9)	0.629

Outcome of matched cohort for NEC or SIP. Mahalanobis distance matching criteria were GA, ICH, PVL, European origin, BPD, female gender, antenatal administration of steroids, and maternal education; p-values are derived from T-Test (IQ), Pearson’s Chi-square test or Mann-Whitney U-test; the type I error level was set to 0.05; data are given as mean (SD) or n (%); significant findings (p < 0.05) are given in bold.

### Growth parameters

In addition, head circumference at the age of 6 years is significantly lower in infants with NEC (48.8 ± 2.0 vs. 50.1 ± 2.1 cm, p = 0.004) and SIP (49.2 ± 2.4 vs. 50.4 ± 2.3 cm, p = 0.011). Infants with NEC history at the age of 6 years present with significant reduced body length as their matched controls (110.0 ± 6.4 vs. 113.0 ± 5.5 cm, p = 0.024).

## Discussion

In a large cohort of VLBWI from the German Neonatal Network, we found that history of NEC but not SIP is associated with an increased risk of impaired neurological development, in particular reduced IQ scores at 6 years of age. Infants with NEC had a three times increased relative risk to develop IQ results <85. So far, most studies on the neurodevelopment of VLBWI reported outcome at the age of 18–36 months^17^ or used data of cohorts from the 1990s^18^ which are not necessarily comparable with recent cohorts who have a decreased short-term morbidity including NEC^19–22^. In one study, the authors found a reduced IQ score in children with a history of gastrointestinal disease (NEC or SIP)^11^. In our study, we made a clear distinction between surgical NEC and surgical SIP based on pathophysiological and macroscopical (as defined by attending surgeon) criteria. The pathophysiology of NEC is based on the immaturity of the gastrointestinal tract and understood as a multifactorial interplay leading to inflammatory processes^23^. Distinct characteristics of bacterial colonization and inappropriate colonization of the premature intestine predispose infants to NEC suggesting a causal relationship between gut bacteria and NEC^24,25^. Together with an inadequate anti-inflammatory response observed in the immature intestine, dysbiosis triggers an inflammatory cascade leading to NEC. Intestinal permeability is observed in NEC and modulated through the expression of Toll-like receptors (TLR4)^12^. Nino *et al*. found a link between NEC and brain injury through activation of TLR4 on microglial cells in the brain. TLR4 stimulation by gut-derived mediators impacts brain injury since TLR4-deficient mice were protected from NEC-induced brain injury. In the setting of prematurity, NEC is associated with a “proinflammatory regulatory protein profile”^15^ which suggests a link between sustained inflammation and adverse neurodevelopmental outcomes^2,14^.

In our matched cohort, we found decreased head circumferences in the NEC and SIP group. Some studies report substantial growth failure (<10^th^ percentile) for weight, length, and head circumference in infants with NEC^26^, but results were conflicting^11^. One study examining head biometrics in magnet resonance imaging (MRI) after NEC found associations with reduced biparietal width^27^. Another study comparing white matter abnormalities (WMA) on brain MRI in NEC and SIP infants showed that infants with NEC had higher WMA scores than those with SIP^28^ assuming a higher vulnerability of oligodendroglial precursors after systemic inflammatory processes such as NEC. In our study, we cannot exactly give an explanation for our findings and can only speculate about causes. It is unclear if our findings are the result of sustained inflammation originating in the neonatal period, the result of chronic malnutrition in this vulnerable population or statistical biased observations. For example, the reduced head circumference in the SIP cohort could be explained by significant reduced birth weight and reduced lower gestational age in the matched SIP group leading to a reduced head growth. We therefore think that our findings concerning growth parameters should be interpreted with caution and further scientific efforts should clarify the role of inflammation or malnutrition in these infants, as interventions here could improve neurologic outcome.

NEC and SIP are both entities with low incidences in a very unique patient cohort. Here, our study has a powerful setting as a large multi-centre study with prospective collection of data and uniform follow-up assessment by the same study team. However, our study also has limitations. We decided to use stringent criteria for NEC (NEC requiring surgery) and SIP (requiring surgery) as primary outcome measure. Hence, the diagnosis of “medical NEC”, i.e. NEC not requiring surgery, which might be difficult to distinguish from other entities, was not considered. Second, our NEC or SIP definition is based on the clinical evaluation of the attending surgeon and neonatologist and not necessarily based on histopathology. Third, our follow-up cohort has a risk of selection bias. For the follow-up, we chose a random invitation practice, but were not able to reduce some differences in both groups. Some factors that were associated with better neurological outcomes were more common in children who were followed-up compared to those who were not including higher birth weight and higher exposure rate with antenatal steroids. On the other hand, BPD and multiple birth were overrepresented in the followed-up cohort. For these differences we accounted with logistic regression models and according matching strategies. Fourth, our study is a post-hoc analysis of an observational population-based design. Hence, a causal relationship cannot be made and mechanistic modelling is necessary. Furthermore, we are not able to rule out the possibility of unrecognized confounders. Preterm birth is associated with several postnatal complications that might impact neurologic development^29^. Potential factors such as antibiotic use, gut dysbiosis, nutrition, use of several drugs or unrecognized systemic reactions might have an impact on neurodevelopmental outcome which we cannot adjust for. Additionally, we were not able to control our analyses for short bowel syndrome as known risk factor for neurological impairments^18^ as we did not record this disease in our follow-up examinations. To account for a variety of known confounders we used Mahalanobis distance matching as it is proved to be a valid approach for adjustments for multiple outcomes^30^. The matched cohort showed a homogenous distribution of factors for NEC which suggest that the model fits adequately. In the SIP group, gestational age and birth weight were significantly lower than in the control group. However, both groups do not differ concerning outcome characteristics, strengthening our conclusion that SIP even at lower GA has no impact on intelligence quotient.

In conclusion, our results reflect that the different aetiology and inflammatory extent of NEC and SIP may lead to disparate neurodevelopment trajectories. Hence our data suggest a potential role of an early gut-brain axis distortion in infants with NEC.

Future longitudinal studies, specifically in cohorts with interventions on the preterm infants microbiome such as PRIMAL^31^, along with detailed mechanistic models are needed to disentangle the impact of gut dysbiosis and sustained inflammation on adverse neurodevelopmental outcome after prematurity.

## Methods

### Ethics

Approval by the local ethic committee for research in human subjects of the University of Lübeck (file number 08–022) and by the local ethic committees of all participating centres has been given. Written informed parental consent was given for the research and publication of the results of each infant included in the study. The German Neonatal Network is funded by the German Ministry for Education and Research (BMBF-grant-No: 01ER0805 and 01ER1501).

### Cohort and definitions

The German Neonatal Network (GNN) is an ongoing multicentre population-based cohort study enrolling VLBWI with <1500 g birth weight in Germany. From 2009 until 2014, 43 tertiary German tertiary level NICUs contributed to the GNN (www.vlbw.de)^32^. Data were collected prospectively by neonatologists or trained study personal. Infants <1500 g birth weight and with a gestational age below 37 weeks were enrolled. A predefined clinical data set including antenatal and postnatal treatment and outcome data was recorded prospectively. The proper assessment of clinical data was ensured by yearly on-site-monitoring by a study nurse or paediatrician experienced in neonatology. NEC requiring surgery is defined as clinical NEC classified as Bell Stage II or Bell Stage III with the need for laparotomy with or without resection of necrotic gut, and the macroscopic diagnosis of NEC^33^. Clinical NEC without surgical treatment was excluded from our analysis. SIP diagnosis was defined as the occurrence of spontaneous intestinal perforation with the need for laparotomy and the macroscopic diagnosis of isolated SIP as described by the attending surgeon. Small for gestational age (SGA) was defined as birth weight <10^th^ percentile according to population based birth weight reference values^34^.

### Six-year follow-up

At the age of 5–6 years, VLBW children were invited for standardized follow-up examination by a study team consisting of a physician and nurses. During the invitation procedure, the study team contacted the clinic of birth for possible follow-up examination dates. The contact database was randomly searched for possible candidates with focus set on infants born <28 weeks gestational age, but infants born >28 weeks were not necessarily excluded. Infants who were born in the clinic of interest, contactable via telephone or postal letter and who could present at the follow-up examination appointment received an invitation letter.

### Motor development

Cerebral palsy (CP) was defined as disorder of the central nervous system characterized by abnormal muscle tone in at least one extremity and abnormal motor development assessed with the Gross Motor Function Classification System (GMFCS ≥ level 1)^35^ and Bimanual Fine Motor Function (BFMF ≥ level 1)^36^. VLBW children without functional limitations were scored as “Level 0”.

### Cognitive development

Total, verbal, and performance intelligence were assessed using the *Wechsler Preschool and Primary Scale of Intelligence* – Third Edition [WPPSI I-III, German]^37^.

### Statistical analysis

#### Cohort characteristics

To describe the characteristics of the whole cohort of VLBWI, we present differences of infants with NEC, SIP or without one of these complications. Data are presented as numbers, frequencies and 95% confidence intervals (CI) of column percentages.

#### Neurological outcome analysis

For neurological outcome analysis at 6 years of age, we included infants who were capable to perform a *Wechsler preschool and primary scale of intelligence* test (WPSSI-III, German edition). Logistic regression analysis and Mahalanobis distance matching were used to calculate a potential impact of NEC or SIP on outcome at six years with simultaneous controlling for several potential confounding factors. Mahalanobis’ method is expected to be very successful in reducing bias in multivariate matching^31^ and was used to compare NEC or SIP affected individuals with unaffected controls with similar risk profile.

#### Logistic regression analysis

Odds ratio and corresponding 95% confidence interval deriving from a logistic regression model was calculated to characterize an association of intelligence quotient <85 at 6 years of age and CP with neonatal complications as potential confounders: birth weight, gestational age (GA), female gender, NEC, SIP, intracerebral haemorrhage (ICH) grade ≥3 and periventricular leukomalacia (PVL), neurosurgery for ventriculoperitoneal shunting (post-haemorrhagic hydrocephalus), European ethnicity, bronchopulmonary dysplasia (BPD), born small for gestational age (SGA), surfactant application, and maternal education level. We calculated the relative risks (RR) derived from the logistic regression by using the proposed method from Zhang *et al*.^16^.

For multiple testing, p-values were adjusted using Bonferroni-Holm method and regarded significant when p < 0.05. Infants who were not capable for WPSSI testing are analysed separately using descriptive statistics.

#### Mahalanobis distance matching

To analyse the effects on intelligence quotient and motor outcome, we matched the participants into two groups for each complication: NEC positive and NEC negative or SIP positive and SIP negative clinical courses. We matched the groups via Mahalanobis distance multi-dimensional modelling^38^. Matching was based on the calculated Mahalanobis distance, including gestational age, ICH, PVL, European ethnicity, BPD, female gender, antenatal administration of steroids, and maternal education for NEC and SIP analysis. For each index case with NEC or SIP, matches were chosen by the best fitting non-affected nearest partner using calculated Chi-squares of two distances.

All statistical analyses were performed with SPSS 22.0 software (IBM SPSS Statistics for Windows, Version 22.0. Munich, Germany). Graphics were created using GraphPad Prism Version 7.00 for Mac (GraphPad Software, La Jolla California USA, www.graphpad.com).

### Ethics approval and consent to participate

Written informed consent was obtained from parents on behalf of the infants enrolled in our study. The study parts were approved by the local committee on research in human subjects of the University of Lübeck (08–022; 03.12.2010) and the local ethical committees at the other study centres.

Specifically: Ethical Board of the Medical Chamber of the North Rhine region, Ethical Board of the University of Aachen, Ethical Board of the University of Bonn, Ethical Board of the Medical Chamber of the federal state of Mecklenburg-Vorpommern, Ethical Board of the Medical Chamber of Berlin, Ethical Board of the University of Magdeburg, Ethical Board of the University of Halle, Ethical Board of the University of Tübingen, Ethical Board of the Medical School Hannover, Ethical Board of the University of Cologne, Ethical Board of the University of Essen, Ethical Board of the Medical Chamber of the Westphalia-Lippe region, Ethical Board of the Medical Chamber of Hamburg, Ethical Board of the Medical Chamber of the federal state of Hessen, Ethical Board of the Medical Chamber of the federal state of Baden-Württemberg, Ethical Board of the Medical Chamber of the federal state of Bavaria, Ethical Board of the Saar University.

### Consent for publication

Consent was given by the parents or legal guardians.

## Supplementary information

Supplementary Information.

## Data Availability

The datasets generated and analyzed during the current study are not publicly available but can be reviewed on reasonable request.
