# Supplementary material for: Surgical necrotizing enterocolitis but not spontaneous intestinal perforation is associated with adverse neurological outcome at school age
Source: Sci Rep. 2020 Feb 11;10:2373. doi: 10.1038/s41598-020-58761-6 (PMC7012917; doi:10.1038/s41598-020-58761-6)
Supplement: Supplementary file 1 — Supplementary Information. [file 41598_2020_58761_MOESM1_ESM.docx]

**Supplementary material**

**Surgical necrotizing enterocolitis but not spontaneous intestinal perforation is associated with adverse neurological outcome at school age**

Alexander HUMBERG, Juliane SPIEGLER, Mats Ingmar FORTMANN, Michael ZEMLIN, Janina MARISSEN, Isabelle SWOBODA, Tanja K. RAUSCH, Egbert HERTING, Wolfgang GÖPEL and Christoph HÄRTEL for the German Neonatal Network (GNN)

| **Characteristics** | **No follow-up at six years**  **n = 5540** | **Follow-up at six years**  **n = 2482** | **Follow-up at six years, no WPPSI testing**  **n = 241** | **p-value ^1)^**  **p-value ^2)^** |
| --- | --- | --- | --- | --- |
| Gestational age (SD) [weeks] | 29.0 (2.8) | 28.9 (3.7) | 27.4 (2.5) | **< 0.001**  **< 0.001** |
| Birth weight (SD) [g] | 1088 (309) | 1163 (677) | 917 (307) | **< 0.001**  **< 0.001** |
| Female Gender | 2719 (49.3) | 1214 (48.9) | 95 (39.4) | 0.770  **0.002** |
| Multiple birth | 1800 (32.6) | 872 (35.1) | 74 (30.7) | **0.027**  **0.021** |
| Antenatal steroids | 4869 (88.9) | 2105 (91.2) | 212 (88.3) | **0.002**  0.092 |
| ICH | 899 (16.4) | 379 (16.4) | 60 (24.9) | 0.999  **< 0.001** |
| PVL | 177 (3.2) | 56 (2.4) | 10 (4.1) | 0.056  0.065 |
| BPD | 791 (14.4) | 414 (17.9) | 71 (29.5) | **< 0.001**  **< 0.001** |
| European origin | 4488 (82.9) | 2080 (90.1) | 207 (87.7) | **< 0.001**  0.190 |
| NEC | 134 (2.4) | 43 (1.8) | 4 (1.7) | 0.096  0.809 |
| SIP | 82 (1.5) | 41 (1.7) | 10 (4.1) | 0.410  **0.003** |

*Supplementary table 1: Baseline characteristics of VLBWI without six-year follow-up, with six-year follow-up and six-year follow-up without WPPSI testing of primary GNN cohort. Data are given as mean (SD) or n (%); p-values are derived from Pearson’s Chi-square test or Mann-Whitney U-test, the type I error level was set to 0.05; percentages are given as column percentages. 1) no follow up vs. follow-up; 2) follow-up vs. no WPPSI testing*

| **Characteristics** | **NEC** | | | **SIP** | | |
| --- | --- | --- | --- | --- | --- | --- |
|  | **No**  n = 43 | **Yes**  n = 43 | **p-value** | **No**  n = 41 | **Yes**  n = 41 | **p-value** |
| Age at follow-up (mean) [months]^¶^ | 71.5 (5.2) | 71.4 (5.3) | 0.776 | 72.0 (4.5) | 70.2 (5.2) | 0.166 |
| Birth weight [g] | 938 (518) | 764 (256) | 0.141 | 917 (309) | 749 (260) | **0.013** |
| Length (birth) [cm] | 34.1 (4.2) | 32.5 (3.9) | 0.107 | 34.7 (4.4) | 32.5 (3.8) | **0.021** |
| Head circumference (birth) [cm] | 24.3 (3.8) | 23.2 (2.5) | 0.095 | 24.8 (2.6) | 23.0 (2.5) | **0.003** |
| Gestational age [weeks] | 27.3 (3.5) | 26.1 (2.2) | 0.154 | 27.7 (2.2) | 25.4 (1.9) | **< 0.001** |
| Female gender | 21 (48.8) | 25 (58.1) | 0.387 | 16 (39.0) | 13 (31.7) | 0.488 |
| Multiple birth | 14 (32.6) | 15 (34.9) | 0.820 | 9 (22.0) | 19 (46.3) | **0.020** |
| Antenatal administration of steroids | 36 (85.7) | 35 (81.4) | 0.591 | 34 (82.9) | 38 (92.7) | 0.177 |
| ICH | 16 (38.1) | 16 (37.2) | 0.933 | 10 (24.4) | 17 (41.5) | 0.100 |
| PVL | 2 (4.8) | 3 (7.0) | 0.664 | 0 | 0 | n.a. |
| BPD | 12 (28.6) | 18 (41.9) | 0.200 | 14 (34.1) | 14 (34.1) | 1.000 |
| Neurosurgery | 0 | 0 | n.a. | 0 | 2 (4.9) | 0.152 |
| Maternal education > 10 years | 18 (42.9) | 16 (37.2) | 0.595 | 13 (33.3) | 18 (46.2) | 0.247 |
| European origin | 33 (78.6) | 35 (83.3) | 0.578 | 34 (82.9) | 31 (75.6) | 0.414 |

*Supplementary table 2: Characteristics of matched VLBWI who were followed-up at six years; Mahalanobis distance matching criteria were GA, ICH, PVL, European origin, BPD, female gender, antenatal administration of steroids, and maternal education; ¶ age corrected for gestational age; p-values are derived from Pearson’s Chi-square test or Mann-Whitney U-test; the type I error level was set to 0.05; data given as mean (SD) or n (%); significant findings are given in bold.*

| **Independent variable** | **Intelligence quotient < 85** | | | **Cerebral palsy** | | |
| --- | --- | --- | --- | --- | --- | --- |
|  | **OR (CI 95%)** | **p-value** | **p-value§** | **OR (CI 95%)** | **p-value** | **p-value§** |
| Birth weight | 1.0 (1.0-1.0) | 0.871 | 1.000 | 1.0 (1.0-1.0) | 0.421 | 1.000 |
| Gestational age (per week) | 0.9 (0.8-1.0) | 0.013 | 0.224 | 0.8 (0.7-1.1) | 0.066 | 1.000 |
| Female gender | 0.7 (0.5-0.9) | 0.016 | 0.256 | 0.7 (0.4-1.1) | 0.120 | 1.000 |
| ICH ≥ grade 3 and PVL | 3.7 (2.3-6.0) | < 0.001 | **< 0.001** | 14.9 (9.0-24.8) | < 0.001 | **< 0.001** |
| Neurosurgery | 3.9 (1.5-10.4) | <0.001 | **0.005** | 3.0 (1.3-6.9) | < 0.001 | 0.003 |
| European origin | 3.3 (2.3-4.8) | < 0.001 | **< 0.001** | 1.7 (0.8-3.3) | 0.226 | 1.000 |
| Administration of surfactant | 1.0 (0.7-1.4) | 0.861 | 1.000 | 1.2 (0.6-2.2) | 0.602 | 1.000 |
| BPD | 2.2 (1.4-3.5) | < 0.001 | **0.015** | 1.7 (1.0-2.9) | 0.020 | 0.411 |
| SGA | 2.2 (1.4-3.5) | 0.001 | **0.019** | 1.4 (0.6-3.3) | 0.431 | 1.000 |
| Maternal education level | 0.4 (0.3-0.6) | < 0.001 | < 0.001 | 1.1 (0.7-1.7) | 0.808 | 1.000 |
| NEC | 4.3 (2.1-8.8) | < 0.001 | **< 0.001** | 1.4 (0.5-4.6) | 0.367 | 1.000 |
| SIP | 1.0 (0.4-2.6) | 0.985 | 1.000 | 1.2 (0.4-3.8) | 0.909 | 1.000 |

*Supplementary table 3: Odds ratios and corresponding 95% confidence intervals deriving from logistic regression analysis (controlled for birth weight, gestational age (per weeks), female gender, ICH ≥ grade 3 and PVL, neurosurgery, European origin, administration of surfactant, BPD, SGA, and maternal education level (coded as maternal school attendance > 10 years)); § Bonferroni-Holm correction (corrected for each model), significant findings (p < 0.05) are given in bold*
